# “Magical relief”: the effectiveness of three stages of a video-based magic intervention on distress and pain in children aged 9–11 years during HPV mass vaccinations—a cluster-randomized trial

**DOI:** 10.1016/j.eclinm.2026.103876

**Published:** 2026-04-18

**Authors:** Anne W.E. Versluis, Edmond H.H.M. Rings, Arno A.W. Roest, Victor Middelkoop, Nienke Vreeken, Andrea W.M. Evers, Henriët van Middendorp

**Affiliations:** a Department of Pediatrics, Willem-Alexander Children’s Hospital, Leiden University Medical Center, Leiden, the Netherlands; b Health, Medical and Neuropsychology Unit, Leiden University, Leiden, the Netherlands; c Center for Interdisciplinary Placebo Studies (IPS) Leiden, Leiden, the Netherlands; d Department of Pediatrics, Sophia Children’s Hospital, Erasmus University Medical Center, Rotterdam, the Netherlands; e Medical Delta, Leiden University, Delft University of Technology, and Erasmus University Rotterdam, the Netherlands

**Keywords:** HPV vaccination, Magic-based intervention, Procedural distress, Pain, Cluster-randomized controlled trial

## Abstract

**Background:**

Vaccinations often elicit significant distress in school-aged children, which can impact pain perception and future medical experiences. This study examined the effects of three stages of a video-based magic intervention on self-reported distress and pain in children receiving HPV vaccinations.

**Methods:**

This cluster-randomized controlled trial randomized 412 children (aged 9–11 years) who received their first HPV vaccination at five mass-vaccination sites in The Netherlands. Based on vaccination date and time, children were assigned to one of four groups: 1. watching a magic trick video during the vaccination, 2. watching the trick with revelation of the secret, 3. watching the trick with revelation of the secret, followed by a brief post-vaccination video-training, and 4. a regular care control. Children completed questionnaires before (T0) and after (T1) the first vaccination in April 2024, and before the second vaccination six months later (T2) in September/October 2024. The primary outcome of child-reported distress was assessed at T0, T1, and T2 using the Facial Image Scale (FIS) and the short form of the State-Trait Anxiety Inventory (STAI-6). The study was preregistered on OSF (https://doi.org/10.17605/OSF.IO/5ASM9, registered April 4, 2024).

**Findings:**

All randomized participants were analyzed according to the intention-to-treat principle. Children in the combined magic-intervention groups (groups 1–3) reported less distress and lower pain after the first vaccination compared to the control group (group 4; distress: *p* < 0.0001, partial *η*^2^ = 0.11; pain: *p* = 0.0039, *η*^2^ = 0.02, 95% CI [0.002–0.058]). The most extensive magic intervention (group 3) showed the largest distress reduction as compared to all other groups (*p* < 0.0001). No significant differences in distress were observed preceding the second vaccination, indicating a lack of sustained effects.

**Interpretation:**

The video-based magic intervention reduced distress and pain immediately following the vaccination, with the largest effect found for the most elaborate intervention group involving active engagement. These findings suggest a promising, easily implementable intervention to improve children’s vaccination experiences during mass vaccinations.

**Funding:**

LUMC Foundation (non-profit).

**Research in context**

**Evidence before this study**

We searched PubMed and Embase for articles published in English up to June 2025, using the terms “vaccination”, “children”, “distress”, “pain”, “distraction”, “magic-based intervention” and “magic”, including synonyms and variant spellings. Vaccinations are among the most common medical procedures in childhood, yet many children fear needles and experience substantial procedural distress. Distress can negatively affect their vaccination experience, may impair cooperation, and shape long-term attitudes towards healthcare. Distraction techniques such as music, videos, and virtual reality have shown promise in reducing distress, but remain underused in mass-vaccination settings and may not fully engage children. Magic-based interventions offer a potentially more immersive form of distraction, with different stages being theorized to bring about additive effects. A small number of randomized controlled trials have demonstrated that magic can reduce self-reported anxiety in children with dental anxiety, and other programs have been effective in alleviating child- and caregiver-reported anxiety in inpatient pediatric units. However, one small trial during influenza vaccination reported no effects on children’s stress response or self-reported pain. To our knowledge, no randomized controlled trial has evaluated a scalable, video-based magic intervention during mass HPV vaccinations.

**Added value of this study**

This cluster-randomized controlled trial is the first to evaluate the effects of different stages of a video-based magic intervention (1. watching a trick, 2. learning the secret behind the trick, and 3. learning to perform the trick) during HPV vaccinations in a large-scale setting. Children receiving the magic interventions reported a significantly greater reduction in distress and pain immediately after vaccination compared to controls, with the largest effects observed in those actively engaged in learning and performing the trick. The one-time intervention was not shown to impact anticipatory distress six months later.

**Implications of all the available evidence**

Active engagement, by learning to perform a magic trick, appears key to reducing distress and pain during vaccinations. The video-based magic intervention is a practical, low-cost, and easily scalable tool with potential applications in vaccination and broader pediatric healthcare settings. Future research should compare video-based interventions with face-to-face approaches, magic interventions with other distraction techniques, explore the variability in children’s responses, and assess feasibility and acceptability from the perspective of healthcare professionals.

## Introduction

Vaccines are a ubiquitous component of pediatric healthcare, offering protection against a wide range of preventable diseases.^1^ Although vaccinations are among the most common medical procedures, the majority of children are afraid of needles, and undergoing vaccinations often elicits high levels of procedural distress.^2^, ^3^, ^4^, ^5^ In fact, children describe the pain and fear of needles as the worst thing about getting vaccinations.^6^

Procedural distress is the multi-dimensional response to unpleasant stimuli, including discomfort, anxiety, fear, and pain.^7^ It manifests through three types of responses: 1) phenomenological (the self-reported anxiety and pain), 2) behavioral (agitated movement, crying), and 3) physiological (increased heart rate). Instantaneously, elevated procedural distress may lead to uncooperative behavior and negatively affect the child’s vaccination experience.^8^ Longer term, this may contribute to negative associations with medical procedures and healthcare in general, anticipatory distress, and reluctance toward future medical treatments, ultimately jeopardizing both individual and public health.^2^^,^^6^^,^^9^, ^10^, ^11^ Despite the growing interest in strategies to mitigate distress during vaccinations, these strategies remain underused in daily practice, particularly in mass-vaccination settings.^12^

Distraction techniques, such as watching videos or the use of Virtual Reality, are among the most commonly studied methods to mitigate distress during medical procedures.^4^^,^^5^ These methods redirect the attention of the child away from the medical procedure, reducing the child’s limited attention to focus on the distress.^4^ These methods are often relatively easy to implement into daily care and do not require specialized training.^4^^,^^5^ However, they may not always fully engage the child.

Another promising distraction method is the use of magic.^13^, ^14^, ^15^, ^16^ Compared to other distraction methods, magic seems to engage children on a deeper cognitive and emotional level, by fostering curiosity and wonder, and is specifically appealing given children’s inherent disposition toward magical thinking.^17^ Besides, magic tricks allow for tailoring to the child’s age and cognitive level and are relatively quick to learn.^15^ Empirical evidence suggests three different underlying stages of magic-based interventions: seeing the trick, learning the secret, and learning to perform the trick.^16^ Seeing the trick predominantly seems to spark curiosity, which distracts from pain or anxiety. By promising the child to learn the secret of the magic trick, positive expectancies and suspense may build, leading to a release of tension after learning the secret of something ‘impossible’.^18^, ^19^, ^20^ Finally, learning to perform the trick can enhance skills, gain a sense of control, and build resilience by being able to do something that others cannot.^13^, ^14^, ^15^^,^^21^

Whereas the use of magic seems promising and several magic-based programs are active in the clinical setting, empirical evidence of their effectiveness is limited.^14^^,^^16^ Only a few randomized controlled trials demonstrated that magic reduced self-reported anxiety in children with dental anxiety.^22^, ^23^, ^24^, ^25^ Other programs were found effective in alleviating child- and caretaker-reported anxiety in inpatient pediatric units.^21^^,^^22^ In contrast, a small study involving 50 healthy children aged 6–11 during routine influenza vaccinations found no significant effects of the performance of magic tricks on the stress response or self-reported pain.^26^ Thus, more evidence is needed, especially for easily implementable magic interventions, as ‘live magic’ often used in previous trials is not feasible in mass-vaccination settings. Therefore, to the best of our knowledge, this is the first study examining the effects of different stages of a video-based magic intervention during mass vaccinations in children.

This study aimed to examine the effectiveness of a magic intervention and different versions thereof (conform the magic stages) in the alleviation of self-reported distress and pain in children receiving their Human Papillomavirus (HPV) vaccinations. It is hypothesized that children in the combined magic intervention groups will show a significantly larger reduction in self-reported distress from before to after the vaccination and lower pain than the control group receiving regular care. Secondary hypotheses were that: 1) each magic stage (magic groups 1–3) contributes to a significant reduction of experienced distress and pain as compared to the control group, with the least distress when learning to perform the trick themselves (stage 3), followed by learning the secret of the magic trick (stage 2) and finally only seeing the trick (stage 1), and 2) the magic interventions lead to lower pre-vaccination distress six months later, right before the second HPV vaccination.

## Methods

### Study design

This cluster-randomized controlled trial was conducted in the Netherlands in collaboration with the Municipal Public Health Service (GGD Hollands Midden). The study took place at four mass-vaccination locations (Hillegom, Katwijk aan Zee, Leiden, and Leiderdorp). T-1 questionnaires were completed either at home prior to arrival or at the vaccination location by the child’s caregiver, T0/T1 occurred at the vaccination locations on 5 afternoons in April 2024, T2 data were collected at the vaccination locations on 4 afternoons/evenings in September/October 2024. The trial is part of the larger “MagicKids” project, and was preregistered prospectively in OSF Registrations (https://doi.org/10.17605/OSF.IO/5ASM9, registered April 4, 2024).^27^ Only variables pertinent to this article are reported; other preregistered variables will be published in a forthcoming paper.

### Participants

All children invited for their first HPV vaccination at one of the selected mass-vaccination locations received a study flyer along with their regular vaccination invitation, which was sent by post by the National Institute for Public Health and Environment (RIVM). Inclusion criteria for children were: 1) attendance at one of the selected locations for their first HPV vaccination, 2) being 9 or 10 years old (11-year-olds were included but excluded from per-protocol analyses), 3) sufficient Dutch or English language skills, and 4) caregiver consent for participation. Children with significant cognitive impairment that prevented self-report (as determined by their caregiver) were excluded. One caregiver per child was eligible for inclusion if they met all of the following criteria: 1) planned to be present during their child’s HPV vaccinations, 2) had sufficient Dutch or English language skills, 3) no other caregiver(s) participated in the study. Only the child’s psychiatric diagnosis (reported at T-1) was used from the caregiver dataset for this paper. Gender data was assessed with a single self-report item: “what is your gender?” (options: boy, girl, and other).

### Randomisation and masking

The study distinguished four groups, corresponding to the three magic stages (1. watching a magic trick video during the vaccination, 2. watching the trick with revelation of the secret, 3. watching the trick with revelation of the secret, followed by a brief post-vaccination video-training), and one control group receiving regular care. To ensure feasibility within a large-scale vaccination setting, clusters were formed by vaccination date and time, using 1–1.5 h time slots. A methodologist who was not involved in the study randomized each timeslot, with all children within a timeslot being assigned to the same group. To reduce a possible location effect, all groups occurred once in Leiden (which was a vaccination location twice). To ensure sufficient power for the primary analysis (combined interventions vs. control), the control group was allocated one extra time. Allocation concealment was ensured using sequentially numbered sealed envelopes, opened 15 min before the start of each new timeslot by AV. Given the nature of the intervention, blinding was not possible.

### Procedures

All invited children or their caregiver(s) who were interested in participation could scan the QR code on the leaflet to leave their email address via Formdesk. They then received the information letters and informed consent forms by email. After providing informed consent via Castor EDC, caregivers reported their relationship to the child, psychiatric diagnoses of the child, the child’s previous vaccination experiences, and their expectations of pain and distress for the current vaccination (T-1, max 2 min). Providing informed consent on paper at the vaccination location was also possible (they received a T0 booklet that also contained the T-1 questions).

Upon arrival for the first HPV vaccination, children received a paper booklet (T0, max 5 min) in which they were asked to report on their current distress, expected distress and pain, experienced control, self-confidence, and resilience. After completion, they were informed about their allocated group and vaccinated. In all three intervention groups, children received a video on a tablet during the vaccination featuring Dutch illusionist Victor Middelkoop (Victor Mids) showing either a magic trick (group 1) or the same trick with secret revelation (group 2 & 3). Videos were matched in duration (1 min and 10 s). In addition to the video, group 3 also received materials for the magic trick and a step-by-step instruction video (± 2 min) after the vaccination, with assistance from the research team if needed. The control group received regular care from the Municipal Public Health Service, typically consisting of conversations with the healthcare professional (e.g., about sports) or comfort from the caregiver (e.g., sitting on the lap). After the vaccination (and for group 3, after the training), children completed the T1 booklet (max 5 min) on current distress, vaccination experiences (e.g., anxiety, pain), and, for the magic-based intervention groups, their experiences with the intervention, experienced control, self-confidence, and resilience.

Six months later, children received a second HPV vaccination invitation by post and a reminder email from the research team. Upon arrival for the second vaccination, children completed the T2 booklet (max 5 min) on current distress, expectations, and for the magic-based intervention groups, their experiences with the intervention that they received last time, experienced control, self-confidence, and resilience. No intervention was provided at T2. Before the vaccination, all children received the magic materials and access to a secured online instruction video (except for children allocated to group 3, who already received them during the previous vaccination).

### Outcomes

The primary outcome of child-reported distress was assessed at T0, T1, and T2 using the Facial Image Scale (FIS)^28^ and the short form of the State-Trait Anxiety Inventory (STAI-6).^29^ The FIS is a validated measure comprising five faces (5 = very unhappy to 1 = very happy), from which children circled the one matching their current feeling. The Dutch version of the STAI-6 is a validated 6-item anxiety scale, rating current feelings (e.g., tense, worried) on a 4-point Likert scale (1 = not at all, 4 = very much) (T0: *α* = 0.856, T1: *α* = 0.770, T2: *α* = 0.822). Due to substantial construct overlap between the measures of the FIS and STAI-6, the primary outcome was the standardized composite distress score derived from the FIS and STAI-6, following the approach of Pravder et al. (2019).^21^ For each instrument and timepoint, baseline (T0) mean and SD were used as reference values to preserve time effects. The composite distress scores at each timepoint were calculated as the unweighted mean of the two standardized scores. Additionally, analyses were performed with the separate scale scores.

As secondary outcome, child-reported pain was measured immediately after the vaccination using a self-developed 0–10 NRS item (“I thought the vaccination hurt”, 0 = not at all, 10 = very much). Distress during vaccination (T1) was calculated by combining two items into one scale (“I was anxious during the vaccination” and “I was stressed during the vaccination”) (*α* = 0.926).

Furthermore, three 0–10 NRS items assessed intervention effects in the intervention groups. At T1, children reported on anxiety (“I think the magic intervention has helped me to be less afraid of the vaccination”), pain (“I think the magic intervention has helped me to experience less pain of the vaccination”), and reluctance towards the vaccination (“I think the magic intervention has helped me to be less reluctant towards the vaccination”) (0 = not at all, 10 = very much). At T2, comparable questions were formulated about the last vaccination (“I think the magic intervention that I received during the last vaccination has helped me to be less afraid/experience less pain/be less reluctant of the vaccination of today”).

An overview of all outcome measures of the larger “MagicKids” study is available in the OSF preregistration.^27^ Expected distress before the vaccination (T0) was calculated by combining two items (“I think I will be anxious during the vaccination” and “I think I will be stressed during the vaccination”) (*α* = 0.856). Expected pain was reported before the vaccination (T0) (“I think the vaccination will hurt”, 0 = not at all, 10 = very much).

### Statistical analysis

Power calculations (G∗Power 3.1.9.4) were based on a comparable RCT that examined the effects of a magic-based intervention in a pediatric ward (Cohen’s *d* = 0.73, *f* = 0.37).^21^ To avoid overestimation from a single study, and to allow comparison across intervention versions, we used a more conservative *f* = 0.15. With *α* = 0.05 and power of 0.80 in a 2 × 2 mixed ANOVA, 90 children per group were required (total 360). To account for a 10% attrition rate, target enrollment was 400.

Data analyses followed the intention-to-treat principle, with supplementary per-protocol analyses. Missing data were not imputed, analyses were conducted using available cases. Internal consistency was assessed using Cronbach’s alpha. Descriptive statistics (age, gender, accompanying caregiver, psychiatric diagnosis) were obtained, and successful randomization was checked using t-tests/ANOVAs for continuous variables, and chi-squared tests for categorical variables. Standardized distress difference scores (T1–T0, T2–T0) were computed to assess changes in primary and secondary outcomes.

For the primary analysis, a Repeated Measures ANOVA (2 × 2) was used to assess distress at T0 and T1 between the combined intervention groups (1–3) and the control group. When a significant interaction effect was found, One-Way ANOVA (using distress change T1-T0) with all pairwise comparisons (using Bonferroni correction) was performed. The same analyses were performed for FIS and STAI-6 separately. A Repeated Measures (2 × 2) ANOVA was also performed to assess distress change between T2 and T0, again followed up by a One-Way ANOVA with distress change T2-T0 in case of a significant interaction between group and time, followed by pairwise comparisons with Bonferroni correction.

All analyses were performed two-sided, with α = 0.05, SPSS version 29. Effect sizes were calculated. Partial *η*^2^ of 0.01 was interpreted as a small effect, 0.06 a medium effect, and 0.14 a large effect. Cohen’s *d* of 0.2 was considered a small effect, 0.5 a medium effect, and 0.8 a large effect.^30^

### Ethics statement

The Medical Research Ethics Committee Leiden-Den Haag-Delft confirmed that the Medical Research Involving Human Subjects Act did not apply to this study (N24.013). Children received an age-appropriate informed consent form, caregivers provided online or on-site written informed consent for both their child’s and their own participation.

### Role of the funding source

The funder of the study had no role in study design, data collection, data analysis, data interpretation, or writing of the report.

## Results

**Fig. 1:**
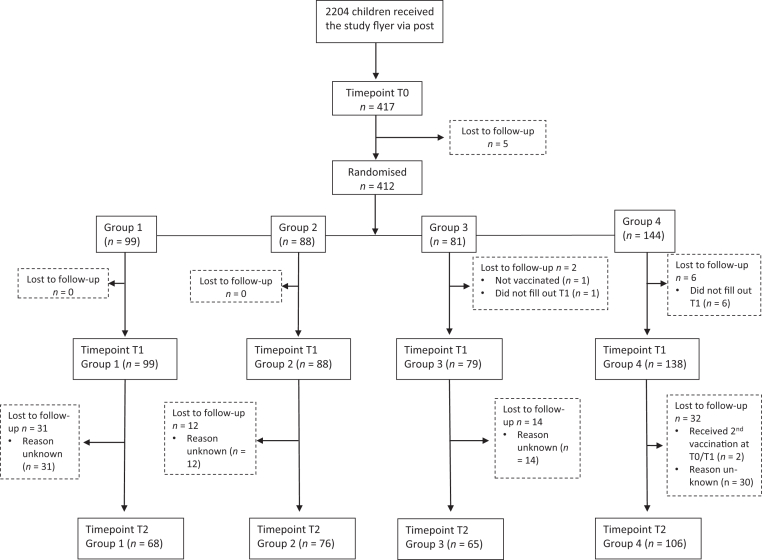
Trial profile. T0 = Before the first HPV vaccination, T1 = after the first HPV vaccination, T2 = 6 months after the first vaccination, right before the second HPV vaccination. Group 1 = only seeing the video of the trick, group 2 = seeing the trick and learning the secret behind the trick, group 3 = seeing the trick, learning the secret and learning to perform the trick, control group = regular care.

In total, 2204 children received the study flyer via post between March 18–24, 2024, of whom 412 children were randomized into the study (group 1: *n* = 99, group 2: *n* = 88, group 3: *n* = 81, control group: *n* = 144), see Fig. 1. The majority of children were 9 years old (*n* = 262, 67%), and the gender distribution was about equal. The location distribution was: Katwijk aan Zee (*n* = 119), Leiden first day (*n* = 105), Leiden second day (*n* = 85), Leiderdorp (*n* = 63), and Hillegom (*n* = 40). The most common accompanying caregiver was the mother (*n* = 294, 75%). Of the seven percent of children with a psychiatric diagnosis (*n* = 27), ADHD was most common (*n* = 16, 59%). Due to ad-hoc missing data, the number of participants differs slightly between analyses. The clinical and sociodemographic characteristics are listed in Table 1.

**Table 1:** Participant characteristics of the intention-to-treat sample.

	All (*n* = 412, 100%)	Group 1 (*n* = 99, 24%)	Group 2 (*n* = 88, 21%)	Group 3 (*n* = 81, 20%)	Group 4 (control) (*n* = 144, 35%)
Age in years, n (%)					
9	262 (67%)	68 (72%)	58 (67%)	46 (60%)	90 (68%)
10	127 (32%)	26 (27%)	28 (33%)	31 (40%)	42 (31%)
11	2 (1%)	1 (1%)	0 (0%)	0 (0%)	1 (1%)
Sex, n (%)					
Boy	192 (49%)	47 (48%)	41 (47%)	33 (43%)	71 (53%)
Girl	201 (51%)	50 (52%)	45 (52%)	44 (57%)	62 (47%)
Other	1 (0%)	0 (0%)	1 (1%)	0 (0%)	0 (0%)
Accompanying caregiver, n (%)					
Mother	294 (75%)	72 (74%)	61 (71%)	56 (72%)	105 (81%)
Father	86 (22%)	22 (23%)	24 (28%)	18 (23%)	22 (17%)
Both/other	11 (3%)	3 (3%)	1 (1%)	4 (5%)	3 (2%)
Psychiatric diagnosis, n (%)					
No	358 (92%)	88 (92%)	83 (98%)	69 (88%)	118 (91%)
Yes	27 (7%)	8 (8%)	2 (2%)	6 (8%)	11 (9%)
I’d rather not say	3 (1%)	0 (0%)	0 (0%)	3 (4%)	0 (0%)
Previous vaccination, n (%)					
Yes	382 (98%)	94 (98%)	86 (100%)	72 (95%)	130 (98%)
No	9 (2%)	2 (2%)	0 (0%)	4 (5%)	3 (2%)
Child’s expected distress (0–10 NRS); mean ± *SD*^a^	4.94 ± 2.98	4.62 ± 2.90	5.05 ± 2.89	5.51 ± 3.29	4.77 ± 2.90
Child’s expected pain (0–10 NRS); mean ± *SD*^b^	4.63 ± 2.86	4.34 ± 2.99	4.61 ± 2.73	5.00 ± 3.05	4.64 ± 2.74

The described *n* is subject to small differences in participant count, as not all participants completed all questions. Group 1 = only seeing the video of the trick, group 2 = seeing the trick and learning the secret behind the trick, group 3 = seeing the trick, learning the secret and learning to perform the trick, control group = regular care.

a Higher scores indicate more expected distress.

b Higher scores indicate more expected pain.

At baseline, no significant group differences were found on demographic characteristics and expected distress and pain. The per-protocol analyses, excluding twelve participants with major deviations from the study protocol (2.9%), showed results that were in line with those of the intention-to-treat sample (see Appendix Tables A1 and A2). Reasons for protocol deviation included not seeing the video in time or not at all despite being assigned to one of the intervention groups, being eleven years old at the time of vaccination, or receiving the second HPV vaccination during the T0/T1 assessments. All assumptions for the proposed analyses were met.

Regarding distress from before (T0) to after (T1) the first vaccination between the combined intervention groups (*n* = 244) and the control group (*n* = 129), a Repeated Measures ANOVA revealed a significant main effect of time, *F*(1,371) = 45.41, *p* < 0.0001, partial *η*^2^ = 0.11, indicating a significantly lower amount of distress across groups at T1 than at T0. Also, a significant interaction effect of time x group, *F*(3,371) = 10.15, *p* = 0.0016, partial *η*^2^ = 0.02, indicating a significantly greater reduction of distress in the combined intervention groups than the control group between T0 and T1. No significant main effect for group was found, *F*(1,371) = 2.95, *p* = 0.0866. A Repeated Measures ANOVA with all four groups also indicated a significant main effect of time, *F*(1,369) = 82.68, *p* < 0.0001, partial *η*^2^ = 0.18, and a significant interaction effect of time x group *F*(3,369) = 4.47, *p* < 0.0001, partial *η*^2^ = 0.08. Again, no main effect of group was found, *F*(3,369) = 1.37, *p* = 0.2514. Comparing distress change (T0-T1) across all four groups using a One-Way ANOVA revealed a significant group difference, *F*(3, 369) = 9.96, *p* < 0.0001, *η*^2^ = 0.08, 95% CI [0.027–0.125], with the most extensive magic-intervention group, which included learning to perform the trick (group 3) (*M* = −1.12, *SD* = 1.20), showing a significantly greater reduction in distress compared to all other groups: group 1 (*M* = −0.41, *SD* = 1.20), group 2 (*M* = −0.44, *SD* = 1.13), and the control group (*M* = −0.23, *SD* = 1.07). All other groups did not significantly differ from each other. The same pattern was found for the separate analyses of the distress between T1 and T0 for the FIS and STAI-6 scales, for the two- and four-group comparisons (see Supplementary Materials). Again, group three showed the largest reduction in both distress measures compared to all other groups in post-hoc comparisons (all *p*-values ≤ 0.0068), see Table 2.

**Table 2:** Child-reported distress and pain in the intention-to-treat sample before the first vaccination (T0), after the first vaccination (T1), and before the second vaccination 6 months later (T2).

	Total	Group 1	Group 2	Group 3	Group 4 (control)
**Before the first vaccination (T0)**	(*n* = 388)	(*n* = 96)	(*n* = 86)	(*n* = 76)	(*n* = 130)
FIS	2.73 ± 0.94	2.63 ± 0.87	2.78 ± 0.93	2.87 ± 1.02	2.68 ± 0.96
STAI-6	13.47 ± 4.33	12.95 ± 4.10	13.33 ± 3.95	14.43 ± 4.73	13.40 ± 4.44
Distress score^a^	−0.00 ± 0.98	−0.17 ± 0.78	−0.02 ± 0.88	0.15 ± 1.01	−0.11 ± 0.88
**After the first vaccination (T1)**	(*n* = 383)	(*n* = 92)	(*n* = 82)	(*n* = 73)	(*n* = 134)
FIS	2.35 ± 1.23	2.29 ± 1.40	2.47 ± 1.20	1.85 ± 1.00	2.59 ± 1.16
STAI-6	11.00 ± 4.02	10.80 ± 4.22	11.09 ± 3.66	9.65 ± 3.70	11.83 ± 4.10
Distress score^a^	−0.48 ± 1.02	−0.53 ± 1.06	−0.42 ± 0.97	−0.90 ± 0.95	−0.26 ± 0.99
Pain	4.59 ± 3.07	4.22 ± 3.02	4.48 ± 3.01	4.08 ± 2.93	5.21 ± 3.16
**Before the second HPV vaccination (T2)**	(*n* = 303)	(*n* = 65)	(*n* = 72)	(*n* = 62)	(*n* = 104)
FIS	2.67 ± 1.05	2.63 ± 0.87	2.78 ± 0.93	2.87 ± 1.02	2.68 ± 0.96
STAI-6	13.37 ± 4.19	12.95 ± 4.10	13.33 ± 3.95	14.43 ± 4.73	13.39 ± 4.44
Distress score^a^	−0.04 ± 0.98	−0.14 ± 0.97	0.07 ± 0.98	0.04 ± 1.16	−0.08 ± 0.90

The described *n* is subject to small differences in participant count, as not all participants completed all questions. Group 1 = only seeing the video of the trick, group 2 = seeing the trick and learning the secret behind the trick, group 3 = seeing the trick, learning the secret and learning to perform the trick, control group = regular care.

a The distress score is a standardized score of the FIS and STAI-6 combined.

**Fig. 2:**
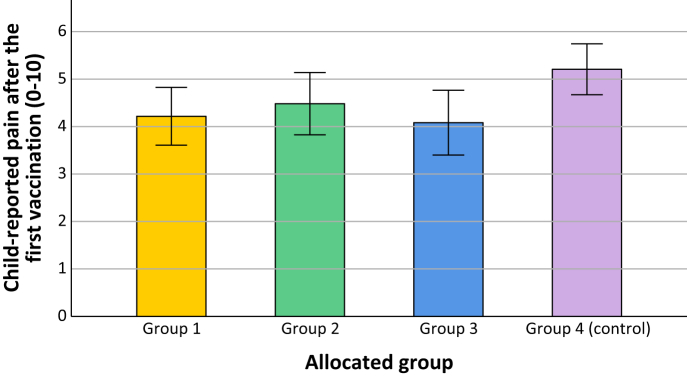
Child-reported pain after the first vaccination (T1) specified per group. Error bars represent 95% confidence intervals. Group 1 = only seeing the video of the trick, group 2 = seeing the trick and learning the secret behind the trick, group 3 = seeing the trick, learning the secret and learning to perform the trick, control group = regular care.

With regard to pain after the vaccination, children in the combined intervention groups reported small-sized significantly less experienced pain (*M* = 4.26, *SD* = 2.98) compared to the control group using a One-Way ANOVA (*M* = 5.21, *SD* = 3.16), *F*(3, 286) = 8.45, *p* = 0.0039, *η*^2^ = 0.02, 95% CI [0.002–0.058]. Comparing all four groups using One-Way ANOVA on pain reported after vaccination showed a significant effect of group (T1), *F*(3, 384) = 3.04, *p* = 0.0289, *η*^2^ = 0.02, 95% CI [0.000–0.054], see Fig. 2. However, even though the lowest pain was reported by the most extensive magic-intervention group and the highest pain by the control group, none of the post-hoc pairwise comparisons reached significance (group 1: *M* = 4.22, *SD* = 3.02; group 2: *M* = 4.48, *SD* = 3.01; group 3: *M* = 4.08, *SD* = 2.92; control group: *M* = 5.21, *SD* = 3.16; all *p*-values ≥ 0.0686, with the difference between pain scores of group 3 and 4 reaching closest to significance).

From T0 (before the first vaccination) to T2 (before the second vaccination), a repeated-measures ANOVA showed no significant change in distress over time (*p* = 0.6640) between the combined intervention groups and the control group, no significant group effect (*p* = 0.3204) and no significant interaction-effect for time x group (*p* = 0.8991). Similarly, no significant effects were found across the four groups (time: *p* = 0.7536, group: *p* = 0.3431, time × group interaction: *p* = 0.6206).

**Fig. 3:**
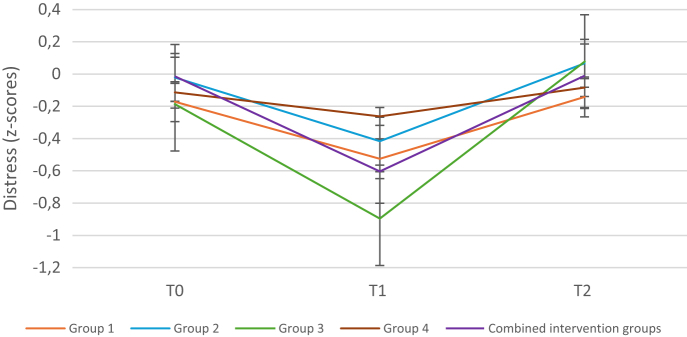
Child-reported course of standardized distress scores per timepoint per group. Group 1 = only seeing the video of the trick, group 2 = seeing the trick and learning the secret behind the trick, group 3 = seeing the trick, learning the secret and learning to perform the trick, control group = regular care.

Separate analyses for the two and four groups of the FIS and the STAI-6 corresponded with these findings. The mean distress scores per group across the three time points are presented in Fig. 3.

In addition to the self-reported experienced distress from before (T0) to after the vaccination (T1), children also reported their experienced distress during the vaccination at T1. No significant differences were found in experienced distress during the vaccination (T1) between the combined intervention groups (*M* = 4.39, *SD* = 3.26) and the control group (*M* = 4.90, *SD* = 3.27), *F*(1,388) = 2.15, *p* = 0.1431, and between all four groups (*p* = 0.4831).

Directly after the first vaccination (T1), children in the magic-intervention groups generally reported on a 0–10 NRS that the magic-based intervention had helped with their anxiety (*M* = 6.64, *SD* = 3.24), with feeling less pain (*M* = 5.94, *SD* = 3.39) and to be less reluctant towards the vaccination (*M* = 6.58, *SD* = 3.17). Before the second vaccination (T2), children in the intervention groups generally still reported that the magic intervention they received during their first vaccination helped them with their anxiety for the current vaccination (*M* = 5.89, *SD* = 3.12, 0–10 NRS), to expect less pain (*M* = 5.28, *SD* = 3.07) and to be less reluctant towards the vaccination (*M* = 4.84, *SD* = 3.22), although these scores were lower than at T1. Whereas children in group 3 consistently reported slightly higher values (i.e., more impact of the intervention) than the other two groups, no significant differences were found between the three intervention groups at T1 and T2 (all *p*-values ≥ 0.1110), see Table 3.

**Table 3:** Child-reported expected effects of the magic-intervention per intervention group.

	Total	Group 1	Group 2	Group 3	*p*-value (ANOVA)
**After the first vaccination (T1)**	(*n* = 234)	(*n* = 83)	(*n* = 80)	(*n* = 71)	
*I think the magic intervention has helped me to …*					
Be less afraid of the vaccination	6.64 ± 3.24	6.73 ± 3.25	6.08 ± 3.33	7.17 ± 3.05	0.1110
Experience less pain	5.94 ± 3.39	5.89 ± 3.41	5.84 ± 3.50	6.11 ± 3.27	0.8725
Be less reluctant towards the vaccination	6.58 ± 3.17	6.22 ± 3.35	6.68 ± 3.20	6.89 ± 2.92	0.4075
**After the second vaccination (T2)**	(*n* = 199)	(*n* = 64)	(*n* = 73)	(*n* = 62)	
*I think the magic intervention that I received during the last vaccination has helped me today to …*					
Be less afraid of the vaccination	5.89 ± 3.12	5.52 ± 2.92	5.66 ± 3.19	6.55 ± 3.18	0.1295
Experience less pain	5.28 ± 3.07	5.17 ± 2.84	4.90 ± 3.16	5.82 ± 3.17	0.2122
Be less reluctant towards the vaccination	4.84 ± 3.22	4.83 ± 3.28	4.78 ± 3.16	4.92 ± 3.27	0.9682

Range 0–10 for all items. Group 1 = only seeing the video of the trick, group 2 = seeing the trick and learning the secret behind the trick, group 3 = seeing the trick, learning the secret and learning to perform the trick, control group = regular care.

## Discussion

This cluster-RCT examined the effectiveness of different versions of a video-based magic intervention (conform the three magic stages) in the alleviation of child-reported distress and pain in children receiving their Human Papillomavirus (HPV) vaccination at mass-vaccination sites in The Netherlands. Overall, children in the combined magic-based intervention groups (1–3), and particularly the most extensive magic intervention group that included magic training, reported a significantly greater reduction in distress and lower pain after the first vaccination compared to the control group. Anticipated distress before the second vaccination, 6 months later, did not differ between groups. Children overall had moderately positive expectations about how the magic intervention helped them during the (preparation for) the vaccination.

The combined magic intervention groups showed a larger decrease in distress from before to after the first vaccination, compared with regular care. This finding is consistent with the hypothesis and with previous studies using face-to-face magic interventions in hospital and dental settings.^21^, ^22^, ^23^, ^24^, ^25^ By contrast, these results were not in line with Teichfischer and colleagues, where no effects of seeing a magic trick during routine vaccination at the pediatricians office on stress response and pain were found in children aged 6–11 years.^26^ A difference between our study and the Teichfischer et al. study was a higher statistical power in the current study, and relatively low baseline stress levels in that study due to familiarity with the physician’s office and physician. Thus, video-based magic interventions might be more effective in more distressing circumstances.

Importantly, the most extensive magic intervention, in which children next to watching and learning the secret behind the trick, also actively learned to perform it, resulted in the greatest reduction in distress and pain. In contrast, merely watching the magic trick or additionally learning its secret was associated with somewhat larger distress-reduction scores, but did not lead to significantly greater reductions in distress or pain compared to regular care. This suggests that active engagement in performing the magic trick could be a key component for relevant distress reduction. Based on the literature, we expected each magic stage to build up in its effects due to different mechanisms being effectuated (from arousing curiosity to tension release and, ultimately, to providing a sense of control and resilience), with learning the trick leading to the most extensive effects as it incorporates all the stages.^16^ To the best of our knowledge, this is the first study to evaluate the effects of each magic stage separately. Our findings suggest that learning to perform the trick is the most essential component in bringing about significant distress-relieving effects. However, a design factor that might provide a (partially) alternative explanation for these findings is that the magic training occurred after the vaccination, which led this group to report on their post-vaccination distress and pain approximately 5 min later than the other groups. Potentially, the mere passage of time could have already led to a decrease. However, considering the trend already seen in the other magic intervention groups towards a decrease in distress and pain, the mere passage of time does not appear to provide the only explanation for the effect. Consequently, based on the current study, the most extensive magic intervention was found to be most promising, but future research needs to uncover how much of this effect is attributable to learning of the trick or to its combination with more general distraction and time effects.

Despite the immediate positive effects on distress, the magic intervention did not impact distress levels preceding the second vaccination, six months later, where all groups returned to baseline levels of distress. This suggests that the brief, one-time magic intervention did not impact general anticipatory distress levels for future vaccination occurrences. This could be due to only having been exposed to a single distress-relieving vaccination experience, which might have been insufficient to achieve lasting changes in distress. Although the magic intervention was generally perceived by children as helpful in reducing anxiety, pain, and reluctance towards the second vaccination, individual opinions varied considerably across all groups. This suggests that factors beyond the intervention itself, or specific factors of the intervention, may have influenced how children valued and responded to the intervention.

An important strength of this study is its high generalizability, as it was conducted with a large sample of children, directly in daily practice during routine vaccinations. The study was conducted in both Dutch and English, enabling participation of children and caregivers with limited or no Dutch language skills. Another strength is the evaluation of both the overall effectiveness of the magic intervention and its effective components, by breaking it into different stages across intervention groups. However, conducting the study in daily practice also posed challenges. A first challenge concerned the timing of T1, as children in group 3 reported their experiences approximately 5 min later than all other groups due to the magic training they received after the vaccination. This additional time effect could have influenced the effect attributable to the magic intervention, as we also expect a natural reduction in distress with time. However, it was neither ethical nor logistically feasible to make all children wait an additional 5 min after the vaccination without purposeful engagement, which could also have introduced additional confounding factors such as boredom. Given these constraints, this approach was the most feasible way to study the intervention within this clinical setting. Another limitation was the reliance on self-report measures. However, children’s experiences are meaningful, as they are expected to impact how children look back at their vaccination experience and, through that, towards future vaccination or more general healthcare experiences. Additionally, the lack of a standardized distraction-based comparison group prevented conclusions on the specificity of magic-effects. Another limitation is that we did not account for clustering in the power analysis, and because individual cluster identifiers were not recorded (only group number and participant location), intraclass correlations could not be estimated post hoc. Furthermore, we were unable to analyze location effects because the vaccination sites differed in capacity and scheduling, resulting in missing several condition x site combinations. To reduce the potential influence of location, the interventions were administered according to a strict protocol by a research team independent of the healthcare providers at each vaccination location. Lastly, the high attrition rate between the first and second vaccination introduced the risk of a self-selected sample. Although the reasons for dropout remain unclear, they may have included scheduling changes (e.g., relocation of the second appointment) or time constraints at the vaccination site, which could limit external validity.

The findings support the notion that active engagement in distraction techniques, such as performing the magic trick, can enhance distress and pain reduction within the vaccination setting. The video-based magic intervention shows potential as a practical, low-cost, and easily implementable tool for healthcare professionals to alleviate distress and pain in children. Future research could compare video-based magic interventions with face-to-face approaches, compare magic interventions to other distraction-based techniques, and assess the feasibility from the perspectives of healthcare professionals. Furthermore, studies should investigate the mechanisms underlying each stage of the intervention to determine whether the proposed processes receive empirical support.

In conclusion, the video-based magic intervention effectively reduced distress and pain in children immediately after the vaccination, with the strongest effects in children who learned to perform the trick themselves. This highlights the importance of active engagement. The brief intervention did not show a lasting effect on pre-vaccination distress six months later, suggesting that multiple successful intervention experiences could be required to achieve lasting effects.

## Contributors

All authors conceptualized the study and developed the protocol. AWEV and HvM, together with a statistician, developed the statistical analysis plan. AWEV and HvM, together with bachelor and master students in psychology at Leiden University, collected the data. The final analysis was performed by AWEV. Both AWEV and HvM accessed and verified the underlying data. AWEV wrote the first draft of the manuscript. All authors had access to all the data reported in the study and had final responsibility for the decision to submit for publication. All authors contributed to and approved the final version of the manuscript and agreed to its submission for publication.

## Data sharing statement

A selection of the data of this study will be available, with restricted access, from the corresponding author (AWEV) upon reasonable request. This only applies for the (pseudo)anonymized data collected through questionnaires.

## Declaration of interests

The authors declare no competing interests. The funder of the study had no role in study design, data collection, data analysis, data interpretation, or writing of the report.

## Appendix A Supplementary data

Supplementary Tables S1–S3

Supplementary Appendices A1 and A2
