# Supplementary material for: “Magical relief”: the effectiveness of three stages of a video-based magic intervention on distress and pain in children aged 9–11 years during HPV mass vaccinations—a cluster-randomized trial
Source: eClinicalMedicine. 2026 Apr 18;95:103876. doi: 10.1016/j.eclinm.2026.103876 (PMC13098333; doi:10.1016/j.eclinm.2026.103876)
Supplement: Supplementary Tables S1–S3 [file mmc1.docx]

**Appendix**

**Table A.1.**

*Participant characteristics of the per-protocol sample.*

| **Characteristics** | **All**  **(*n* = 400, 100%)** | **Group 1**  **(*n* = 97, 24%)** | | | **Group 2**  **(*n* = 85,**  **21%)** | | **Group 3**  **(*n* = 76, 19%)** | **Group 4**  **(control)**  **(*n* = 142, 36%)** |
| --- | --- | --- | --- | --- | --- | --- | --- | --- |
| Age in years, *n* (%)  Nine  Ten | 262 (67%)  127 (33%) | 68 (72%)  26 (28%) | | | | 58 (67%)  28 (33%) | 46 (60%)  31 (40%) | 90 (68%)  42 (32%) |
| Sex, *n* (%)  Boy  Girl  Other | 188 (49%)  194 (51%)  1 (0%) | 46 (48%)  49 (52%)  0 (0%) | | 39 (47%)  44 (52%)  1 (1%) | | | 32 (44%)  41 (56%)  0 (0%) | 71 (54%)  60 (46%)  0 (0%) |
| Accompanying caregiver, *n* (%)  Mother  Father  Both/other | 288 (76%)  82 (22%)  9 (2%) | 71 (75%)  21 (22%)  3 (3%) | | 58 (70%)  24 (29%)  1 (1%) | | | 55 (75%)  16 (22%)  2 (3%) | 104 (81%)  21 (17%)  3 (2%) |
| Psychiatric diagnosis, yes, *n* (%)  No  Yes  I’d rather not say | 346 (92%)  27 (7%)  3 (1%) | 86 (91%)  8 (9%)  0 (0%) | | 80 (98%)  2 (2%)  0 (0%) | | | 64 (88%)  6 (8%)  3 (4%) | 116 (91%)  11 (9%)  0 (0%) |
| Previous vaccination, *n* (%)  Yes  No | 372 (98%)  9 (2%) | 92 (98%)  2 (2%) | 83 (100%)  0 (0.0%) | | | | 69 (94%)  4 (6%) | 128 (98%)  3 (2%) |
| Child’s expected distress (0-10 NRS); mean ± SD^a^ | 4.96 ± 2.97 | 4.68 ± 2.89 | | 5.07 ± 2.87 | | | 5.70 ± 3.26 | 4.69 ± 2.86 |
| Child’s expected pain (0-10 NRS); mean ± SD^b^ | 4.64 ± 2.87 | 4.40 ± 2.99 | | 4.61 ± 2.74 | | | 5.03 ± 3.08 | 4.61 ± 2.74 |

*Note.* The described *n* is subject to small differences in participant count, as not all participants completed all questions. ^a^ Higher scores indicate more expected distress. ^b^ Higher scores indicate more expected pain. Group 1 = only seeing the video of the trick, group 2 = seeing the trick and learning the secret behind the trick, group 3 = seeing the trick, learning the secret and learning to perform the trick, control group = regular care.

**Table A.2.**

*Child-reported distress and pain in the per-protocol sample before the vaccination (T0), after the vaccination (T1), and 6-months later before the second vaccination (T2).*

|  | **Total** | **Group 1** | **Group 2** | | **Group 3** | **Group 4**  **(control)** |
| --- | --- | --- | --- | --- | --- | --- |
| **Before the vaccination (T0)** | (*n* = 380) | (*n* = 95) | | (*n* = 83) | (*n* = 73) | (*n* = 129) |
| FIS | 2.73 ± 0.94 | 2.65 ± 0.86 | | 2.77 ± 0.93 | 2.90 ± 1.02 | 2.65 ± 0.94 |
| STAI-6 | 13.48 ± 4.29 | 13.02 ± 4.08 | | 13.28 ± 3.90 | 14.66 ± 4.73 | 13.27 ± 4.35 |
| Distress score^a^ | -0.00 ± 0.92 | -0.14 ± 0.76 | | -0.04 ± 0.88 | 0.17 ± 1.02 | -0.14 ± 0.84 |
|  |  |  | |  |  |  |
| **After the first vaccination (T1)** | (*n* = 379) | (*n* = 95) | | (*n* = 80) | (*n* = 71) | (*n* = 133) |
| FIS | 2.34 ± 1.16 | 2.20 ± 1.09 | | 2.50 ± 1.21 | 1.86 ± 1.02 | 2.59 ± 1.17 |
| STAI-6 | 11.01 ± 4.04 | 10.82 ± 4.22 | | 11.17 ± 3.69 | 9.61 ± 3.75 | 11.78 ± 4.10 |
| Distress score^a^ | -0.47 ± 1.03 | -0.51 ± 1.07 | | -0.39 ± 0.97 | -0.89 ± 0.96 | -0.26 ± 0.99 |
| Pain | 4.63 ± 3.07 | 4.26 ± 3.02 | | 4.57 ± 3.01 | 4.11 ± 2.95 | 5.20 ± 3.15 |
| **Before the second HPV vaccination (T2)** | (*n* = 294) | (*n* = 63) | | (*n* = 69) | (*n* = 59) | (*n* = 103) |
| FIS | 2.68 ± 1.05 | 2.64 ± 1.01 | | 2.78 ± 1.01 | 2.71 ± 1.25 | 2.61 ± .92 |
| STAI-6 | 13.37 ± 4.23 | 12.95 ± 4.06 | | 13.69 ± 4.20 | 14.02 ± 4.76 | 13.04 ± 4.03 |
| Distress score^a^ | -0.04 ± 0.99 | -0.12 ± 0.98 | | 0.07 ± 0.99 | 0.05 ± 1.17 | -0.10 ± 0.90 |

*Note.* The described *n* is subject to small differences in participant count, as not all participants completed all questions. ^a^ The distress score is a standardized score of the FIS and STAI-6 combined. Group 1 = only seeing the video of the trick, group 2 = seeing the trick and learning the secret behind the trick, group 3 = seeing the trick, learning the secret and learning to perform the trick themselves, control group = regular care.
