# Supplementary material for: “Magical relief”: the effectiveness of three stages of a video-based magic intervention on distress and pain in children aged 9–11 years during HPV mass vaccinations—a cluster-randomized trial
Source: eClinicalMedicine. 2026 Apr 18;95:103876. doi: 10.1016/j.eclinm.2026.103876 (PMC13098333; doi:10.1016/j.eclinm.2026.103876)
Supplement: Supplementary Appendices A1 and A2 [file mmc2.docx]

Supplementary materials

**Supplementary Table 1.**

*Assignment of children by vaccination location.*

|  | **Katwijk aan Zee** | **Leiden**  **(first day)** | **Leiden**  **(second day)** | **Leiderdorp** | **Hillegom** |
| --- | --- | --- | --- | --- | --- |
| Group 1 | 28 | 36 | 34 | 0 | 0 |
| Group 2 | 29 | 0 | 30 | 30 | 0 |
| Group 3 | 24 | 32 | 0 | 0 | 25 |
| Group 4 (control) | 38 | 32 | 24 | 33 | 15 |

*Note.* Values are counts of children per assigned group and vaccination location.

**Supplementary Table 2**

*Repeated Measures 2x2 ANOVA of FIS and STAI-S from T0 to T1.*

| **Measure** | **Effect** | **df1** | **df2** | ***F*** | ***p*** | **Partial *η*²** |
| --- | --- | --- | --- | --- | --- | --- |
| FIS | Time | 1 | 368.97 | 16.38 | < .001 | .04 |
|  | Group | 1 | 378 | 2.83 | .093 | n.s. |
|  | Time x group | 1 | 378 | 8.90 | .003 | .02 |
| STAI | Time | 1 | 373 | 77.97 | < .001 | .04 |
|  | Group | 1 | 373 | 2.68 | .103 | n.s. |
|  | Time x group | 1 | 373 | 7.47 | .007 | .02 |

*Note.* Greenhouse-Geisser corrections were applied when appropriate. n.s. = not significant.

**Supplementary Table 3**

*Repeated Measures 2x4 ANOVA of FIS and STAI-S from T0 to T1.*

| **Measure** | **Effect** | **df1** | **df2** | ***F*** | ***p*** | **Partial *η*²** |
| --- | --- | --- | --- | --- | --- | --- |
| FIS | Time | 1 | 376 | 37.15 | < .001 | .09 |
|  | Group | 3 | 376 | 1.95 | .121 | n.s. |
|  | Time x group | 3 | 376 | 7.74 | < .001 | .06 |
| STAI | Time | 1 | 371 | 120.79 | < .001 | .25 |
|  | Group | 3 | 371 | 1.00 | .393 | n.s. |
|  | Time x group | 3 | 371 | 8.10 | < .001 | .06 |

*Note.* Greenhouse-Geisser corrections were applied when appropriate. n.s. = not significant.
